# Effect of Adalimumab on Refractory Arthritis in Juvenile Idiopathic Inflammatory Myopathy with Anti-MDA5 Autoantibody

**DOI:** 10.1155/2018/2164312

**Published:** 2018-02-06

**Authors:** Takako Miyamae, Takuma Hara, Aki Hanaya, Yumi Tani, Takayuki Kishi, Hisashi Yamanaka

**Affiliations:** ^1^Institute of Rheumatology, Tokyo Women's Medical University, 10-22 Kawada-cho, Shinjuku-ku, Tokyo 162-0054, Japan; ^2^Department of Pediatrics, Tokyo Women's Medical University, 8-1 Kawada-cho, Shinjuku-ku, Tokyo 162-8666, Japan

## Abstract

A 10-year-old girl manifested persistent fever, skin rash, leg pain, fatigue, and joint pain. Based on muscle weakness, elevated muscle-derived enzymes, magnetic resonance imaging, and skin biopsy results, the diagnosis was juvenile idiopathic inflammatory myopathies (JIIM). Chest CT was normal; the anti-melanoma differentiation-associated protein-5 (anti-MDA5) autoantibody was positive. Initial manifestations subsided after prednisolone (PSL) and methotrexate treatment. After the PSL dosage was decreased, the patient presented with metacarpophalangeal (MCP) joint pain and swelling in both index fingers, synovial fluid, and signals on power Doppler ultrasound. The arthritis was refractory to cyclosporine and tacrolimus. Radiography showed progressive MCP joint space narrowing and joint erosion. Adalimumab was initiated 14 months after disease onset. There was a mildly increased matrix metalloproteinase-3 (MMP3) level, an erythrocyte sedimentation ratio (ESR), and a normal CRP level. Adalimumab resulted in decreased MCP joint pain and swelling. PSL was discontinued 10 months after adalimumab initiation; after 9 more months of adalimumab, there were no significant ultrasonography findings. MMP3 and ESR levels normalized during treatment. Radiography after 2 years of adalimumab showed further progressive MCP joint space narrowing restricting dorsiflexion. This report clarified that anti-MDA5-positive JIIM joint manifestations were due to active synovitis and that adalimumab is required for severe cases. Further experience is needed to determine the pathology, severity, and prognosis of this type of arthritis.

## 1. Introduction

The juvenile idiopathic inflammatory myopathies (JIIMs) are heterogeneous, systemic, autoimmune diseases with onset in childhood; they are characterized by weakness, chronic inflammation of the skeletal muscles, and typical skin rashes. Recently, new classification criteria for JIIMs have been developed [1], along with an increased understanding of the spectrum of phenotypes associated with JIIMs based on clinicopathologic features and the presence of myositis-specific autoantibodies (MSAs) and myositis-associated autoantibodies (MAAs) [2, 3]. The MAAs seen most frequently in JIIMs are anti-p155/140, anti-MJ, and anti-melanoma differentiation-associated protein-5 (anti-MDA5) autoantibodies [3].

Anti-MDA5 autoantibodies were present in 33% of a Japanese cohort but in only 7% of a UK cohort with JIIMs [4, 5]. Interstitial lung disease was more common in patients from both cohorts with this autoantibody compared with patients without these antibodies. In the UK patients, other common features included oral and cutaneous ulcerations, arthritis, and milder muscle disease, which were similar to findings in the US and European adult cohorts with anti-MDA5 autoantibodies.

With reference to joint manifestations seen among patients with JIIM and anti-MDA5 autoantibodies, the pathology and prognosis, including the response to medical treatments for myositis, have not been well described. We report the case of a girl with JIIM presenting with anti-MDA5 autoantibodies who had arthritis refractory to conventional treatments and improvement after adalimumab treatment.

## 2. Case Presentation

The patient, a 10-year-old Japanese girl, presented with fever and a skin rash after a sunburn due to daily outdoor swimming in July 2013. However, the fever persisted for two months, and she developed multiple symptoms: general fatigue; a skin rash; joint pain in her wrists, elbows, and knees; and pain in the lower extremities. The skin manifestations on the neck, face, upper extremities, and dorsum of the hands were photosensitivity consisting of Gottron's papules and a heliotrope rash, findings consistent with juvenile dermatomyositis. She was diagnosed as having JIIM based on clinical muscle weakness (manual muscle testing-8 score: 68 (range 0–80)), mildly elevated levels of muscle-derived enzymes (creatine kinase, 196 U/L; aldolase, 13.6 IU), the findings on muscle MR imaging, slightly high-signal intensity in the thigh with T2-weighted fat suppression, and skin biopsy. A muscle biopsy was not performed. The patient did not have respiratory symptoms, and a chest CT revealed no abnormality suggesting interstitial lung disease (ILD), though anti-MDA5 autoantibody was positively detected by an immunoprecipitation assay. Hyperferritinemia (773.9 ng/mL) was noted. Daily doses of 75 mg (1.9 mg/kg) of prednisolone (PSL) and weekly doses of 20 mg of oral methotrexate (MTX) were initiated, after which the symptoms improved. Subsequently, the patient visited our institute after 4 months of observation during the corticosteroid taper when 28 mg (0.7 mg/kg) of PSL was given. The Childhood Myositis Assessment Scale (CMAS) was 50 (range of score: 0–52), and a residual skin rash on the dorsum of the hands was seen. No other organ involvement was observed.

**Figure 1:**
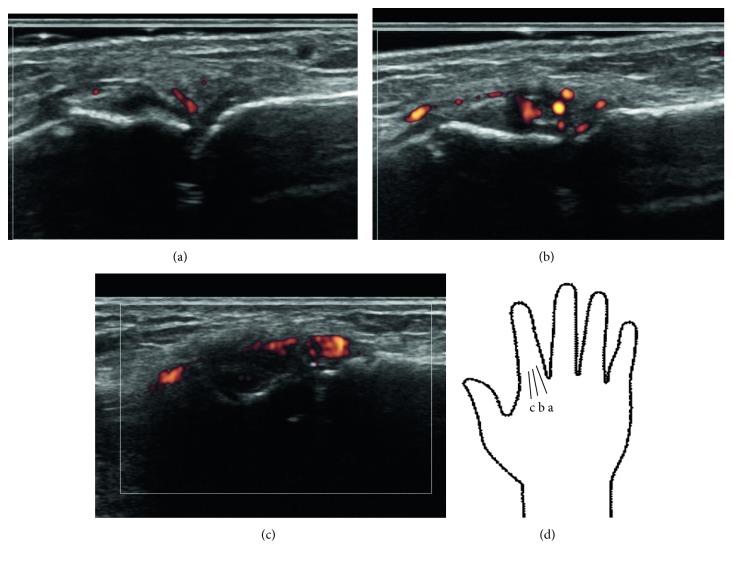
Ultrasonography of the metacarpophalangeal joint of the right index finger. No remarkable findings were seen on a midline echo (a), but synovial fluid and power Doppler signals were noted on the radial-shifted view (b) and (c).

**Figure 2:**
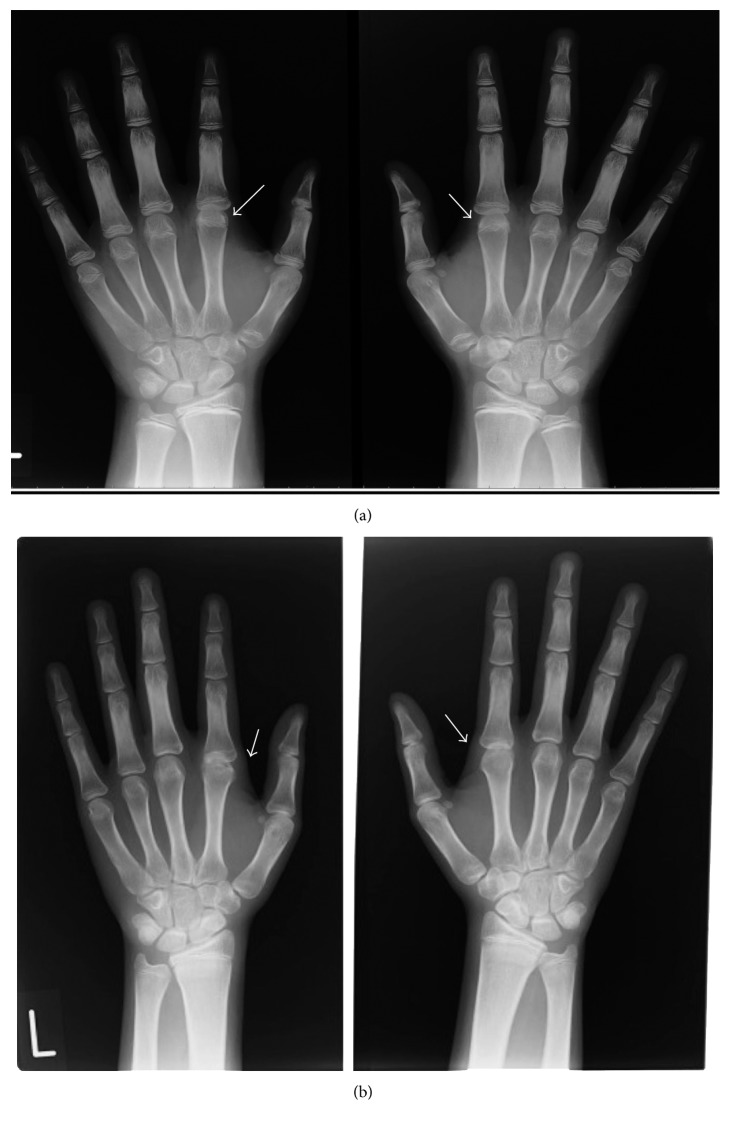
Radiography of both hands. (a) In September 2014, at the initiation of adalimumab when the patient was 12 years and 1 month of age. (b) In September 2016, after 2 years of adalimumab treatment when the patient was 14 years and 1 month of age. Narrowing of the metacarpophalangeal joints of both index fingers was observed when adalimumab was started (a), and more progressive joint space narrowing existed under treatment with adalimumab, whereas insignificant bone erosion was observed (b).

**Figure 3:**
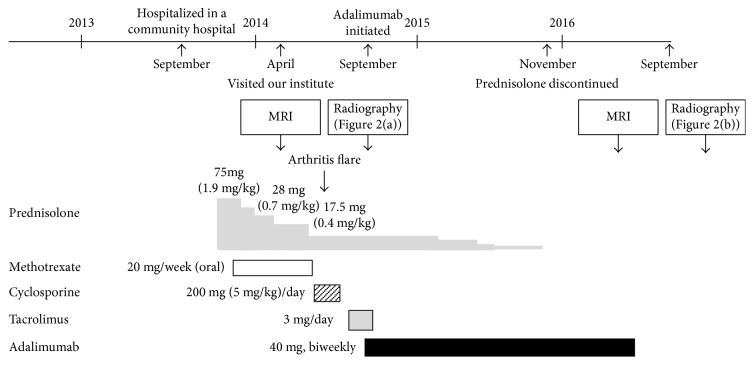
Time course of clinical treatments.

Because osteonecrosis of the right talus and both tibia occurred due to the administration of high doses of glucocorticoids, a further taper of PSL was encouraged. MTX was discontinued after 5 months of administration and changed to 200 mg (5 mg/kg)/day of cyclosporine due to the possibility of MTX-induced ILD and the probability of developing anti-MDA5 autoantibody-associated ILD [6]. When the dosage of PSL was decreased to 17.5 mg (0.4 mg/kg)/day, the patient presented with pain and swelling of the metacarpophalangeal (MCP) joints of both index fingers. Ultrasonography of the MCP joints indicated synovial fluid and power Doppler signals (Figure 1). Antinuclear antibody, rheumatoid factor, and anti-citrullinated peptide antibody were not detected. Because the arthritis was refractory to another disease-modifying antirheumatic drug (3 mg/day of tacrolimus) and radiography showed progressive narrowing of the joint space of the MCP joints (Figure 2(a)), biweekly administration of 40 mg of adalimumab was initiated in September 2014, 14 months after the initial onset of JIIM when the patient was 12 years old. Laboratory data showed a mildly increased level of matrix metalloproteinase-3 (MMP3) of 105.5 ng/mL, an erythrocyte sedimentation ratio (ESR) of 21 mm/hour, and a normal CRP level (0.08 mg/dL). Adalimumab treatment resulted in the subsidence of pain and swelling of the MCP joints. Oral PSL was discontinued 10 months after initiation of adalimumab, and no significant fluid or power Doppler signals were observed on ultrasonography of the MCP joints performed 19 months after the drug's initiation (Figure 3). The elevated levels of MMP3 and ESR were gradually normalized during the time course of adalimumab administration without clinical manifestations of active arthritis, such as swelling and pain. However, radiography in September 2016, after 2 years of adalimumab treatment, showed further progressive narrowing of the MCP joints that restricted dorsiflexion, whereas bone erosion was insignificant (Figure 2). Bone erosion was not detected even on an MRI performed almost at the same time as radiography. Myositis and skin manifestations remained stable during adalimumab treatment.

Anti-MDA5 autoantibody levels were measured by qualitative immunoprecipitation assay. It remains unclear whether a correlation exists between anti-MDA5 autoantibody levels and arthritis activity.

## 3. Discussion

This is the first time that adalimumab was used to treat arthritis associated with JIIM and anti-MDA5 autoantibodies. The initial high doses of corticosteroids prevented the development of ILD and CNS disease but resulted in osteonecrosis. In a series of 54 Japanese patients with JDM, 33% had anti-MDA5 autoantibodies [4], whereas these autoantibodies were present in only 7% of patients in the UK JDM registry [5]. It is also called “an arthro-dermato-pulmonary syndrome” with anti-MDA5 autoantibodies because there was a high rate of interstitial lung disease, arthritis, and distinctive skin lesions, such as ivory-centered papules and/or cutaneous ulcerations, which could develop among the patients [7].

With reference to the joint involvement seen in children with JIIM and anti-MDA5 autoantibodies, few reports have been described so far. In a US cohort study of 160 adult DM patients, anti-MDA5-positive patients were frequently observed with arthritis that was often symmetric, present in the small joints of the hands, and associated with morning stiffness that was clinically similar to rheumatoid arthritis [8]. In the 9 cases with arthritis, a small erosion on the radial site of the 2nd metacarpal head was seen in one patient, but only on MRI of the hands; conventional X-rays did not demonstrate erosive disease in any of the patients.

With regard to joint involvement, the patient in our report met the International League of Associations for Rheumatology (ILAR) classification of juvenile idiopathic arthritis (JIA) [9]. However, the joint manifestation seen in our patient was characteristically limited to the symmetrical small MCP joints of the hands, though the ultrasonography findings were consistent with active synovitis observed in typical JIA. Compared with progressive joint space narrowing on radiography, insignificant bone erosion might be specific to arthritis associated with the disorder. The disease activity of the patient's dermatitis and myositis was relatively mild, and based on our experience, immunosuppressive treatments were mainly required for the refractory arthritis. Considering this patient's joint manifestations, the change in ultrasonography findings, and the time course of the ESR and MMP3 levels, adalimumab apparently contributed to the subsidence of the refractory arthritis and the discontinuation of PSL in this adolescent girl. This case report clarified that the joint manifestations associated with anti-MDA5 autoantibody-positive JIIM were due to active synovitis, although the affected joints were limited to the MCP joints of the index fingers. Although discontinued in this case, MTX is used as a first line immunosuppressant in several treatment algorithms for juvenile dermatomyositis and could be effective against the arthritis independent of PSL [10]. However, this report also indicates the importance of adalimumab treatment in severe cases. Further experience is needed to determine the pathology and prognosis of this type of arthritis.

## Abbreviations

JIIM: Juvenile idiopathic inflammatory myopathy
MSA: Myositis-specific autoantibody
MAA: Myositis-associated autoantibodies
MDA-5: Melanoma differentiation-associated protein-5
PSL: Prednisolone
MTX: Methotrexate
MCP: Metacarpophalangeal
MMP-3: Metalloproteinase-3
ESR: Erythrocyte sedimentation ratio
JIA: Juvenile idiopathic arthritis.
